# Visual expertise modulates resting-state brain network dynamics in radiologists: a degree centrality analysis

**DOI:** 10.3389/fnins.2023.1152619

**Published:** 2023-05-17

**Authors:** Hongmei Wang, Renhuan Yao, Xiaoyan Zhang, Chao Chen, Jia Wu, Minghao Dong, Chenwang Jin

**Affiliations:** ^1^Department of Radiology, First Affiliated Hospital of Xi'an, Jiaotong University, Xi'an, China; ^2^Department of Medical Imaging, Inner Mongolia People's Hospital, Hohhot, China; ^3^Department of Nuclear Medicine, Inner Mongolia People's Hospital, Hohhot, China; ^4^Engineering Research Center of Molecular and Neuro Imaging of Ministry of Education, School of Life Science and Technology, Xidian University, Xi'an, China; ^5^PLA Funding Payment Center, Beijing, China; ^6^School of Foreign Languages, Northwestern Polytechnical University, Xi'an, Shaanxi, China; ^7^Xi'an Key Laboratory of Intelligent Sensing and Regulation of Trans-Scale Life Information, School of Life Science and Technology, Xidian University, Xi'an, China

**Keywords:** degree centrality, visual expertise, object recognition, support vector machine, radiologist

## Abstract

Visual expertise reflects accumulated experience in reviewing domain-specific images and has been shown to modulate brain function in task-specific functional magnetic resonance imaging studies. However, little is known about how visual experience modulates resting-state brain network dynamics. To explore this, we recruited 22 radiology interns and 22 matched healthy controls and used resting-state functional magnetic resonance imaging (rs-fMRI) and the degree centrality (DC) method to investigate changes in brain network dynamics. Our results revealed significant differences in DC between the RI and control group in brain regions associated with visual processing, decision making, memory, attention control, and working memory. Using a recursive feature elimination-support vector machine algorithm, we achieved a classification accuracy of 88.64%. Our findings suggest that visual experience modulates resting-state brain network dynamics in radiologists and provide new insights into the neural mechanisms of visual expertise.

## Introduction

Visual expertise is a cognitive process that involves visual object recognition ability in a specific domain, resulting in superior visual object recognition performance (Harel, 2016; Wang et al., 2021). The development of visual expertise is thought to involve reciprocal interactions between the visual system and multiple high-level areas across the brain (Harel, 2016). In particular, visual expertise is essential for the development of radiological expertise, which enables radiologists to rapidly and accurately recognize abnormalities in medical images (Haller and Radue, 2005; Harley et al., 2009; Hendee, 2010; Melo et al., 2011). However, the neural mechanisms underlying visual expertise in radiology remain poorly understood, particularly with regard to resting-state brain network dynamics. Resting-state functional magnetic resonance imaging (rs-fMRI) can be used to investigate the intrinsic activity of multiple neural networks simultaneously and may help uncover the neural basis of visual expertise in radiology. In this study, we used rs-fMRI to investigate how visual expertise induced by experience modulates the dynamics of brain networks in radiologists.

Previous neuroimaging studies have investigated the neural mechanisms underlying visual expertise using different expertise models. Martens et al. (2018) found that bird expertise-related neural changes involved both low-level and high-level visual regions as well as frontal lobe areas, suggesting that expertise can modulate neural correlates that are specific to the domain as well as those that are more general. Similarly, research on London taxi drivers by Spiers and Maguire (2006) revealed widespread patterns of activation along visual pathways and other brain regions such as the parahippocampal cortex, retrosplenial cortex, and prefrontal structures, indicating their association with scene processing, navigation, and spatial processing when participants inspected landmark objects in city scenes. In the context of radiological expertise, previous studies have reported selective activations in the brain regions of radiologists such as the bilateral middle frontal gyrus (MFG) and left superior frontal gyrus (SFG), which are linked to visual attention and memory retrieval, when comparing brain responses to radiological images between radiologists and laypersons (Haller and Radue, 2005). Furthermore, it was found that the fusiform face area (FFA) was more active when radiologists viewed domain-related images and contributed to the recognition of normal anatomical features based on subjective similarity rather than physical similarity (Harley et al., 2009). This finding was supported by Bilalic et al. (2016) who showed that FFA could help radiologists discriminate X-ray stimuli from other stimuli and then contribute to the evaluation of radiographic images. Lastly, Wang et al. (2021) proposed that visual experience could modulate the functional adaptation of the visual cortex and other cognitive areas that are responsible for decision making, semantic knowledge, and attention, as evidenced by widely altered functional connectivity in the entire cortex including the SFG, MFG, orbitofrontal cortex (OFC), and fusiform gyrus (FuG). Collectively, these studies suggest that the activation of these circuits or brain areas constitutes a cortical organizing principle of visual expertise in the brain, such as visual processing, attention control, decision making, and semantic memory.

Radiology is a particularly suitable domain for investigating the impact of visual experience on expertise because it allows for a comparison between experienced radiologists or medical interns and lay persons who lack experience, enabling the identification of distinguishing traits (Bilalic et al., 2016). Functional magnetic resonance imaging (fMRI) is a promising method to uncover functional adaptations in the entire brain cortex associated with visual expertise. Resting-state brain activity refers to the intrinsic response of the brain in the absence of thinking activity (Smitha et al., 2017; Canario et al., 2021) and the observed brain activity is regarded as being responsible for coding prior experience (Albert et al., 2009; Dong et al., 2014). However, few studies have utilized resting-state fMRI (rs-fMRI) to investigate the neural mechanisms of visual expertise in radiologists. Degree centrality (DC) is a graph-based measurement that can reveal the network dynamics modified by prior experience and node centrality for visual expertise (Reynolds et al., 2018; Liu and Lai, 2022). A support vector machine (SVM) is a machine learning-based pattern classification approach that has unique advantages in understanding small sample learning problems and has been widely applied in biological data processing (Cherkassky, 1997; Li et al., 2014; Liu et al., 2014). The most discriminatory parts of the brain based on SVM represent the most striking feature between the two groups and reveal underlying expertise-related neurobiology (Ding et al., 2015; Gao et al., 2022). By utilizing rs-fMRI, DC, and SVM, we aim to gain a deeper understanding of the neural mechanisms of visual expertise in radiologists.

The main goal of this study was to explore how visual experience modulates DC in resting-state activity and to understand the neural correlates of visual expertise using a model of radiologists (*n* = 22) and rs-fMRI. The DC method combined with a novel but sensitive machine learning method, i.e., a recursive feature elimination-support vector machine (RFE-SVM) (Ding et al., 2015), was employed to look for the highest discriminative power between the radiology intern (RI) group and the normal control (NC) group. We expect that visual experience modulates the expertise-related brain areas beyond the visual cortex and even other cognitive areas, thus supporting working memory (WM), memory, attention control, and decision making (Harel et al., 2013; Harel, 2016; Wang et al., 2021).

## Materials and methods

All study procedures were approved by the Subcommittee on Human Studies of the First Affiliated Hospital of Medical College in Xi'an Jiaotong University and were conducted in accordance with the Declaration of Helsinki.

### Participants

Twenty-two radiology interns and 22 matched subjects were recruited in our study. All of the subjects in the RI group were undergraduates majoring in radiology who interned at the First Affiliated Hospital of Xi'an Jiaotong University. Before rotation, all of the participants received basic medical education at their college. The RI group had X-ray department rotation experience, mainly in interpreting X-ray images for 4 weeks, during which time they practiced 6 days per week and read 25–35 cases per day. The total length of training was 26 ± 2.4 (mean ± standard deviation, SD) days. Scrutinizing the images displayed on the screen and completing the X-ray reports were the main tasks of every intern's training. Each of the interns had a senior tutor providing basic clinical support. After 4 weeks of rotation, at least 600 reports written by each RI were recorded in the Picture Archiving and Communication System (PACS), which were modified by the instructor to meet the “degree of agreement” requirements. The subjects in the NC group were from other majors and had never participated in any form of medical imaging training nor received any related education. The average ages of the RI group and NC group were 23 ± 0.7 years and 23 ± 0.5 years, respectively. The sex distribution in the two groups was the same (11 males; 11 females). The recruitment criteria of all subjects included the following: (1) the participants were physically healthy and right-handed; (2) the subjects and their immediate family members had no past or present neurological, psychiatric, or neuropsychological disorders and had no history of head trauma or brain tumor by medical history, physical, and neurological examinations; and (3) participants took no relevant drugs before or during the internship. Written consent forms were obtained from all the participants.

### Behavioral measurement

Both the RI group and NC group completed the same behavioral tasks. We conducted the prescreening tasks using a face-to-face questionnaire to exclude confounding factors, such as visual expertise from other domains (e.g., cars, chess, birds, and mushrooms). The subjects' behavioral test of the visual expertise level was restricted to X-ray films because of the high specialty for required perceptual expertise (Nakashima et al., 2015). Participants in the RI group were required to pass a practical examination about radiological anatomy and interpretation of X-ray films to verify that they had reached a required level of expertise. The Cambridge Face Memory Test (CFMT) and Radiological Expertise Task (RET) were employed to measure face expertise and radiological expertise in our study. The RET consists of 100 standard X-ray images of adults including 65 positive images and 35 negative images, from the PACS of the X-ray image bank under the guidance of three senior independent expert radiologists with more than 10 years of radiological experience and who were proficient in reading X-ray images. The three senior experts not only scrutinized the pathological appearance of the selected films and confirmed the approval of the reports but also evaluated the level of difficulty of the reports on a scale of 1–3. Sixty-five positive X-ray images contained one nodule without any other conclusions in the corresponding reports and 35 negative images were normal X-rays without any lesions. The level of difficulty for grades 1–3 in all 100 images used in the RET accounted for 55%, 30%, and 15% of the images, respectively. The detailed procedures of CFMT and RET were introduced in our previous research (Zhang et al., 2022).

### MRI data acquisition

fMRI data were collected from 8:30 a.m. to 12:30 a.m. to eliminate the time-of-day effect (Hasler et al., 2014). Brain imaging scans were performed on a 3T GE scanner (EXCITE; General Electric; Milwaukee; Wisc.) at the imaging center of Xi'an Jiaotong University First Affiliated Hospital. A standard birdcage head coil and restraining foam pads were used to minimize head motion and protect participants' hearing. Resting-state functional images were acquired by an echo-planar-imaging sequence, and the specific parameters included 32 contiguous slices with a slice thickness = 4 mm, layer interval = 0, TR = 2,000 ms, TE = 30 ms, FA = 90°, FOV = 240 mm × 240 mm, data matrix = 64 × 64, voxel size = 3.75 mm × 3.75 mm × 4 mm, total volumes = 190, and scanning time = 380s. During the entire resting process, the subjects had to keep their eyes closed, stay awake, and try to keep their minds blank without having any particular thoughts. Additionally, an MPRAGE T1-magnetization high resolution anatomical image (1 × 1 × 1 mm) was acquired for each participant with the following parameters: TE = 2.26 ms, TR = 1,900 ms, flip angle = 9°, FOV = 256 mm, slice thickness = 1 mm, and matrix = 256 × 256. A total of 176 slices in the sagittal orientation were acquired. Potential clinical abnormalities of each participant were assessed by two expert radiologists based on the structural images. No participants were excluded at this level.

### Resting-state fMRI preprocessing

Statistical Parametric Mapping (SPM12, http://www.fil.ion.ucl.ac.uk/spm) and the Data Processing Assistant for Resting-State fMRI (DPARSF 4.5, http://rfmri.org/DPARSF) were used for MRI data preprocessing. The preprocessing steps were as follows: (1) DICOM data were converted to NIFTI format; (2) the first 10 time points were removed for stability of the magnetic field and to allow the subjects to adapt to the experimental environment; (3) slice time correction was conducted for the remaining time points; (4) motion correction was carried out using rigid body transformation to fix the brain at the same target position; (5) the functional images were coregistered to the subject's anatomical images, and all the processed data were divided into gray matter, white matter, and cerebrospinal fluid by the exponentiated lie algebra (DARTEL) tool (Ashburner, 2007); (6) a higher-level Friston-24 model was employed to regress out head motion; (7) the nuisances such as global signal, white matter signal, and cerebrospinal fluid signal were regressed; (8) all the resting functional images were normalized to MNI space using the deformation field maps obtained from structural image segmentation; (9) the normalized fMRI data were resampled to 3 mm isotropic voxels; (10) the images were then spatially smoothed with a 6 mm full width at half maximum (FWHM) Gaussian kernel; and (11) linear trend removal and temporal bandpass filtering (0.01–0.08 Hz) were performed to reduce the effect of low-frequency drifts and high-frequency noise.

### Feature extraction

#### Generation of voxel wise and region wise DC maps

The DC index has unique superiority (i.e., high sensitivity, specificity, and reliability) in reflecting the dynamics of brain networks (Zuo and Xing, 2014). In current study, the DC method was employed to look for the neuroimaging features between groups. The specific steps were as follows: first, the BOLD time course of each voxel was extracted and its Pearson's correlation with all other voxels in the whole brain was analyzed. Every voxel with positive correlation coefficients >0.2 was selected, which can eliminate the weak correlation due to signal noise to ensure that voxels have higher regional functional connectivity strength values. Fisher's *r*-to-*z* transformation was conducted to derive the *Z* score matrix and improve normality for the resulting voxel for each participant. Then, the DC value of each subject was divided by the mean of the whole brain to achieve standardization, which can eliminate individual differences. The DC map of the whole brain based on the voxel-level data was obtained. After that, the voxel wise DC map was averaged into a region wise DC map. The Brainnetome atlas was employed to divide the DC map into 246 regions of interest (ROIs) (Fan et al., 2016). The DC values of all the ROIs were averaged to obtain the average DC value of each region. Finally, the mean DC values from the 246 ROIs then served as the input vector for the classification procedure.

### Feature selection

**Figure 1:**
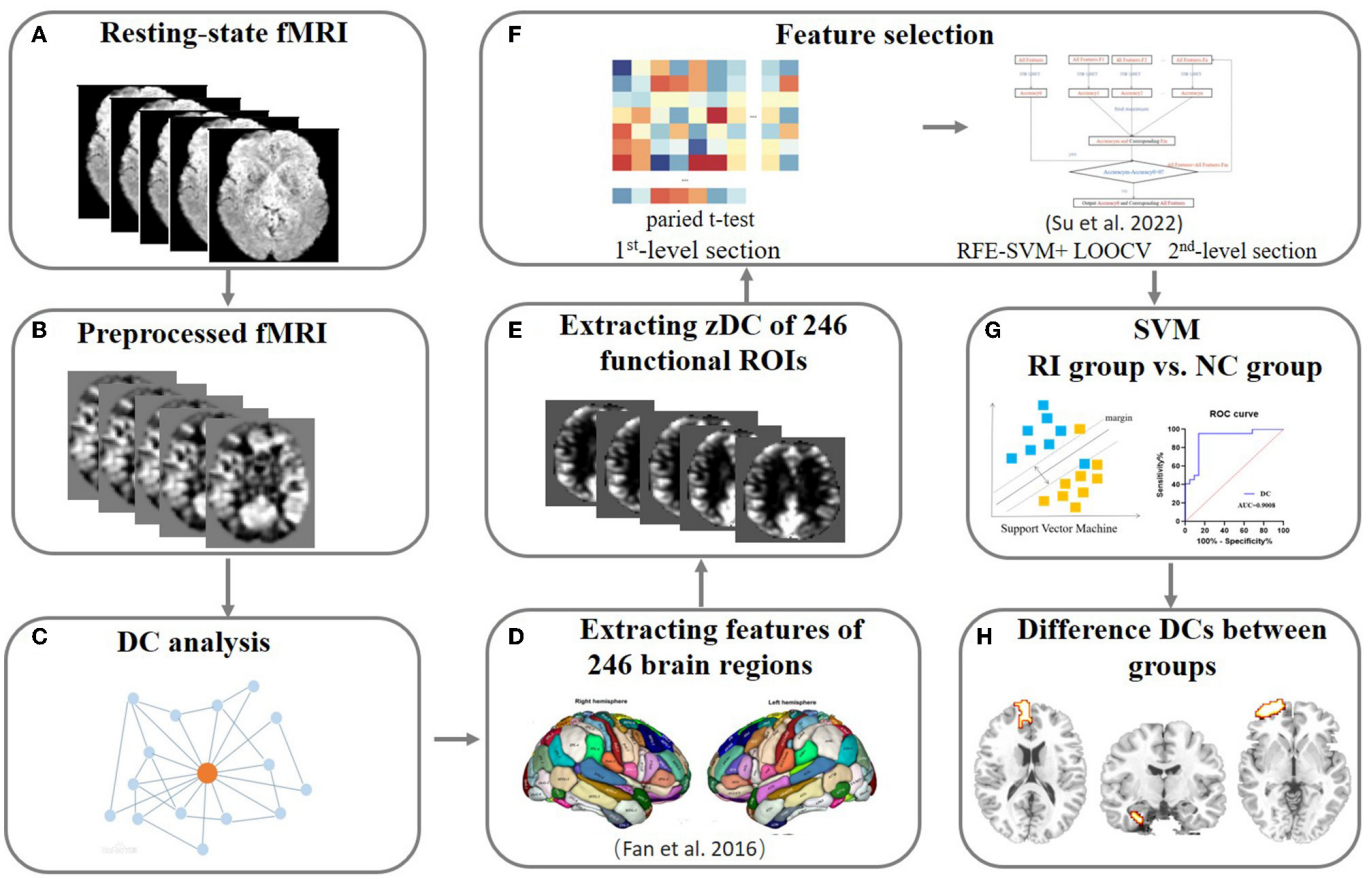
The pipeline of the rs-MRI data analysis. **(A–C)** The resting-state MRI data were collected and preprocessed following procedures described in the Methods. Then, the DC for each voxel was calculated and used for future feature selection. **(D–F)** Feature selection. Two-step feature selection was performed and the first level used a two-sample approach to perform the regional average feature. Then, RFE-SVM modeling with LOOCV was employed to search for the most remarkable features between groups. **(G, H)** SVM modeling. Reliable SVM classification results and the brain areas with robust differences in DC values between groups were obtained to reflect the alteration of dynamics in the whole-brain network. rs-MRI, resting-state MRI; fMRI, functional magnetic resonance imaging; DC, degree centrality; RFE-SVM, recursive feature elimination-support vector machine; SVM, support vector machine; LOOCV, leave-one-out cross-validation.

Feature selection is a hotspot in bioinformatics and is critical in medical studies. Its process is to extract informative features from complex high-dimensional data (Du et al., 2017). We performed a two-stage feature selection procedure in our study. Firstly, we used a two-sample t test to identify the differences in the region wise DC maps between the two groups in a leave-one-out fashion, with a threshold of *p* < 0.05 considered significant. The resulting region wise features were then used in the second-level elimination. In our study, the recursive feature elimination-support vector machine (RFE-SVM) Guyon et al. (2002) is employed for the purpose of feature selection that combines recursive feature elimination with SVM modeling. Basically, we used the RFE-SVM approach in a leave-one-out cross-validation (LOOCV) framework to recursively eliminate the least useful features until further elimination resulted in reduced accuracy. The basic idea behind RFE-SVM is introduced as follows: in each iteration, the contribution to classification accuracy is determined by eliminating one feature at a time using SVM-LOOCV. Then, the features with zero contribution to classification accuracy is taken away from feature set which is to be used as input for next round of iteration. These steps are repeated until the number of features reaches zero. The feature set with highest classification accuracy is used as the outcome of RFE-SVM and sent to SVM for modeling. For this step, we used several performance indicators, including accuracy, sensitivity, specificity, receiver operating characteristic (ROC) curve, and area under the ROC curve (AUC), to evaluate the efficiency of the RFE-SVM classifier. LOOCV was also used to validate the model. Note that a linear SVM classifier model with a soft interval separation and hinge loss function, as it is commonly used in neuroimaging data and produces interpretable results (Rasmussen et al., 2011). The pipeline of rs-MRI data and feature selection processing in this study is illustrated in Figure 1.

### Correlation analysis

To evaluate the relationship between behavioral measurements and the dynamics of the resting brain network in the two groups, voxel wise Pearson's correlation analysis was conducted between the averaged DC values and outcome of behavioral tasks (i.e., RET scores and response times). The significance level was set at *p* < 0.05 after multiple comparison correction (false discovery rate, FDR).

## Results

There were no significant differences in age or sex between the groups (*p* > 0.05). The mean practice level duration and cases in the total RI group are shown in Table 1.

**Table 1:** Demographic data of the radiological intern group and normal control group.

**Labels**	**Radiologists (*n* = 22) Mean ±SD**	**Controls (*n* = 22) Mean ±SD**	***p-*values**
Length of training	26 ± 2.4	N/A	–
Cases in total	767.4 ± 82.6	N/A	–
RET^*^^∧^	0.80 ± 0.04	0.53 ± 0.04	< 0.001
Response time of RET (s)^*^	2.6 ± 0.4	3.7 ± 0.7 s	< 0.001
Face expertise	56.95 ± 5.23	58.68 ± 5.31	0.28

* Denotes significant difference between groups (p < 0.001).

∧ Denotes that the Mann–Whitney test was used.

SD, standard deviation; s, seconds; RET, radiological expertise task; CFMT, Cambridge face memory test.

### Results of behavioral tests

**Figure 2:**
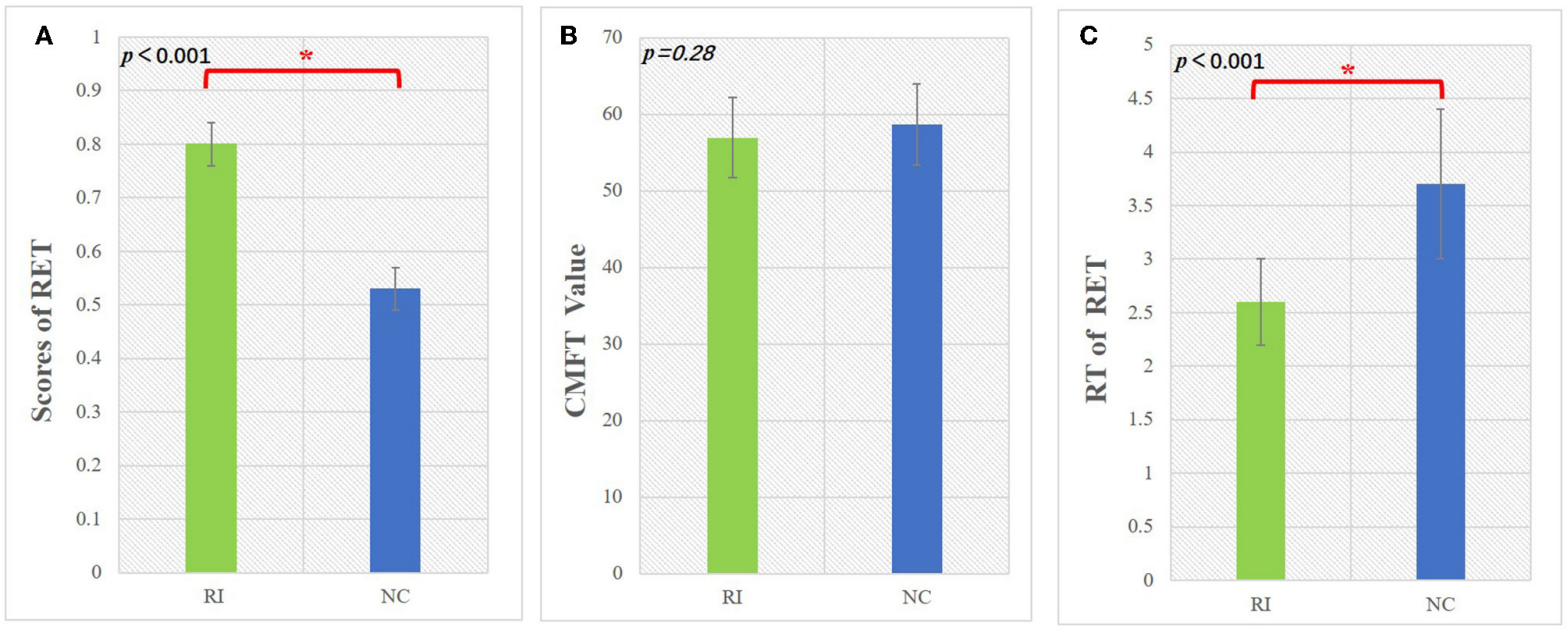
The performance of the behavioral test in the RI group and NC group. **(A)** The scores of lung nodule identification for the two groups. **(B)** The different values in the CFMT for each group. **(C)** The response time of recognizing the lung nodule for each group. RET, radiological expertise task; CFMT, Cambridge face memory test; RT, response time; RI, radiology intern; NC, normal control. ^*^Indicates significant group differences (*p*<0.05).

The behavioral performance of the RI and NC groups is summarized in Figure 2 and Table 1. Compared with the NC group, the RI group had significantly higher RET scores, indicating that the visual experience enabled the RI group to have better nodule recognition ability than the NC group (*p* < 0.001, Mann–Whitney *U*-test). The response time of RET in the RI group was much shorter than that in the NC group, suggesting that the RI group can recognize nodules much faster than the NC group (*p* < 0.001, Mann–Whitney *U*-test). There was no significant difference in CMFT scores between the two groups, which demonstrated that the two groups had similar face recognition abilities (*p* > 0.05, Mann–Whitney *U*-Test).

### SVM classification results

**Figure 3:**
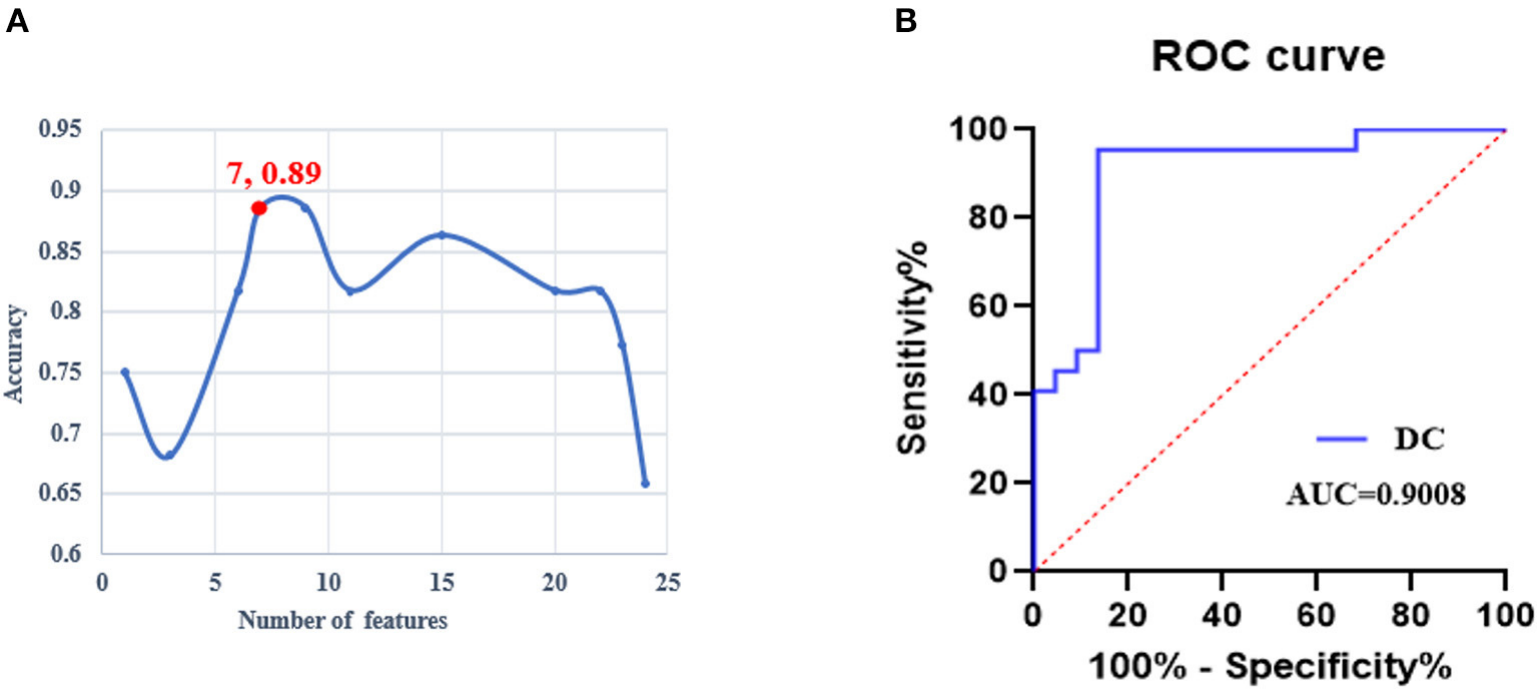
The performance of the RFE-SVM classifier. **(A)** The iteration procedure of feature selection based on RFE-SVM. **(B)** The receiver operating characteristic curve of the RFE-SVM classifier. ROC, receiver operator characteristic; DC, degree centrality; AUC, area under the curve; RFE-SVM, recursive feature elimination support vector machine.

**Figure 4:**
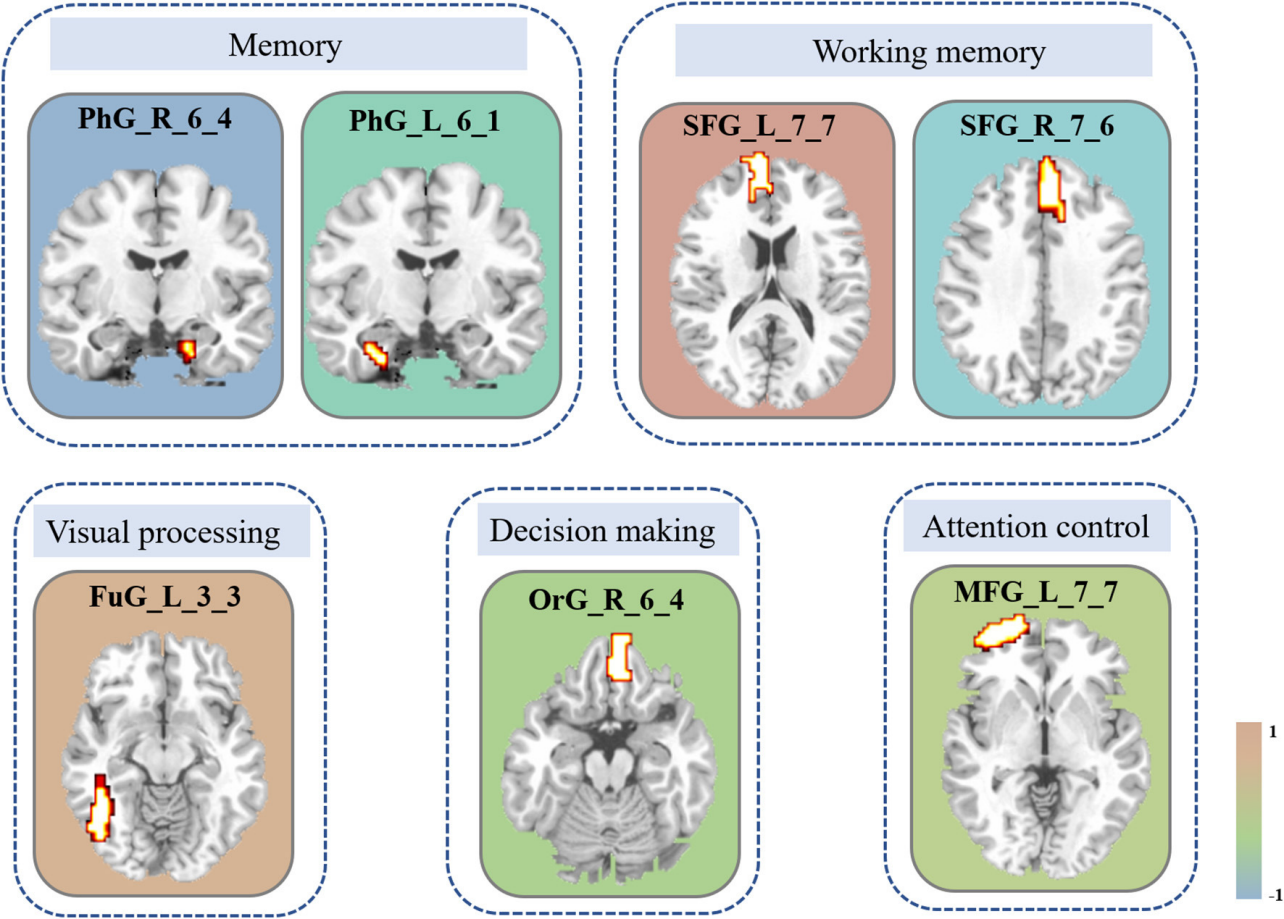
Brain areas with the most discriminative ability between groups. The constitutional diagram is categorized by visual and cognitive components. The color bar shows the size of the weight. Note that the positive direction represents the increased DC values and vice versa. MFG, middle frontal gyrus; OrG, orbital gyrus; FuG, fusiform gyrus; PhG, parahippocampal gyrus; SFG, superior frontal gyrus; DC, degree centrality.

The iteration procedure of feature selection based on RFE-SVM is presented in Figure 3A. The highest classification accuracy was observed in the seventh subset. The brain regions with discriminative power included the bilateral SFG, left MFG, right orbital gyrus (OrG), left FuG, and bilateral parahippocampal gyrus (PhG). The details of the brain regions are shown in Table 2 and Figure 4. The SVM classification accuracy was 88.64%, sensitivity was 81.82%, specificity was 95.45%, and AUC was 0.9008. The ROC curve of classification accuracy for RFE-SVM is presented in Figure 3B.

**Table 2:** The difference in DC values between the RI and NC groups.

**Cognitive component**	**Brain region**	**Subregions**	**Brodmann's areas**	**Side**	**Weight**
Attention control	MFG	MFG_L_7_7	BA10 (lateral)	L	−0.59
Decision making	OrG	OrG_R_6_4	BA11(medial)	R	−0.60
Visual processing	FuG	FuG_L_3_3	BA37 (ventral and lateral)	L	0.56
Memory	PhG	PhG_L_6_1	BA35/36	L	−0.63
		PhG_R_6_4	A28/34	R	−0.99
Working memory	SFG	SFG_R_7_6	BA9 (medial)	R	−0.66
		SFG_L_7_7	BA10 (medial)	L	0.64

BA, brodmann area; FuG, fusiform gyrus; MFG, middle frontal gyrus; OrG, orbital gyrus; PhG, parahippocampal gyrus; SFG, superior frontal gyrus; L, left; R, right.

### Results of correlation analysis

**Figure 5:**
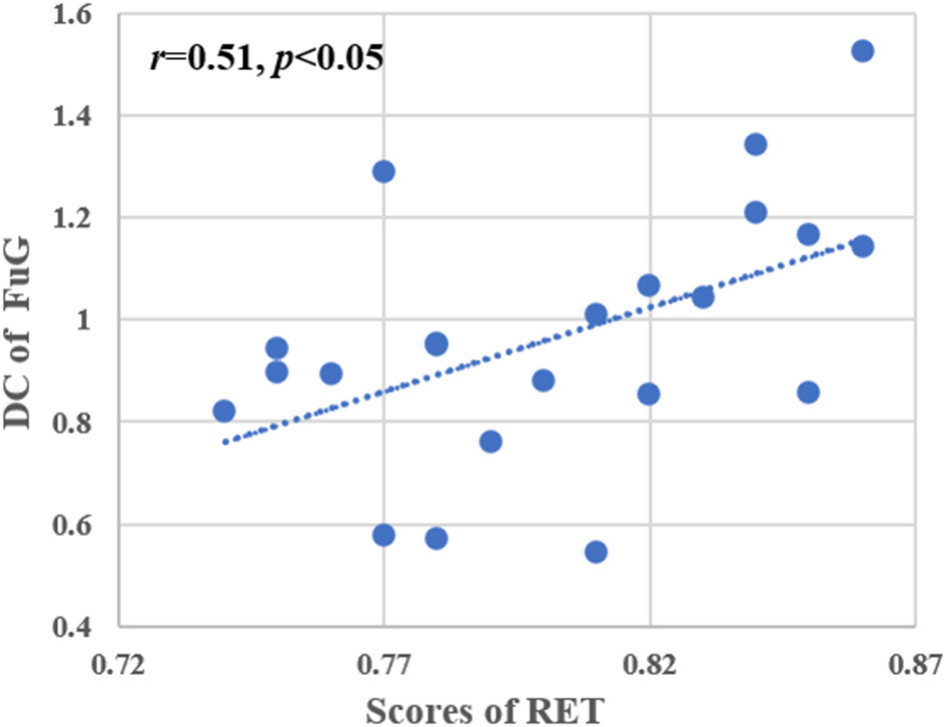
Correlation analysis between RET scores and DC values of the left FuG. Pearson correlation was used to assess significance (*p* < 0.05, multiple comparison corrected). RET, radiological expertise task; FuG, fusiform gyrus; DC, degree centrality.

A significant positive correlation between the average voxel wise DC of the left FuG and the level of radiological expertise (i.e., RET scores) was found in the RI group after multiple comparisons (*r* = 0.51, *p* < 0.05, Figure 5). No significant correlations were found between other indicators of the behavioral tests and DC in the RI or the NC groups.

## Discussion

Visual expertise is a complex skill that requires learning from a vast amount of domain-specific visual information (Dong et al., 2022). Several studies have examined the neural mechanisms underlying radiologists' expertise, identifying high-order cognitive and low-order visual factors such as visual processing, WM, attention control, and decision making as crucial components (Donovan and Litchfield, 2013; Harel, 2016; Annis and Palmeri, 2019). However, the extent to which visual experience modulates resting-state brain activity in radiologists remains unclear. This study aimed to address this gap by investigating how real-world visual experience affects the DC values of resting-state brain activity in radiologists. Our behavioral results showed that the RI group performed better after training than the NC group (Figure 2), and the imaging data analysis demonstrated that seven brain subregions in the visual cortex, prefrontal lobe, and limbic system had the highest discriminative power in between-group comparisons (Figure 4 and Table 2). These results were obtained using RFE-SVM, which demonstrated excellent classification efficiency with high accuracy, sensitivity, and specificity (Figures 3A, B). Additionally, we found a significant positive correlation between RET scores and the DC values of the left FuG, indicating that the functional connectivity of this region is related to visual expertise (Figure 5). To our knowledge, this study is the first to investigate DC level changes in radiologists' resting brains in response to real-world visual experience. The results provide new insights into the neural mechanisms underlying visual expertise, and the findings may have practical applications for radiologist training. Overall, our study highlights the importance of considering resting-state brain activity in understanding how visual expertise develops and may help inform future research in this area.

### The increased DC level of the left FuG in radiologists

Compared with the NC group, the RI group had increased DC values in the left FuG which controls visual processing (Figure 4 and Table 2). Additionally, we found a significant positive correlation between the DC value of the left FuG and RET scores in the RI group (Figure 5). The acquisition of visual experience may be accompanied by functional enhancement of visual processing supporting radiologists' superior performance (Haller and Radue, 2005; Wang et al., 2021). The FuG, located in the human ventral temporal cortex (VTC), is a pivotal functional brain module within the high-level visual cortex (Weiner and Zilles, 2016) which is a key-structure in high-level visual processing for object recognition (Grill-Spector et al., 2001). The FuG contains several category-selective regions for the recognition of different visual stimuli, including the FFA (Kanwisher et al., 1997), fusiform body area (Peelen and Downing, 2005), and visual word-form area (Cohen et al., 2000). Several neuroimaging studies using visual expertise models, such as cars (McGugin et al., 2015), chess (Bilalić, 2016), faces (Goold and Meng, 2017), and radiology (Haller and Radue, 2005) reported activation of the FuG in task fMRI studies. Specifically, the right FuG was engaged in the processing of non-face expertise visual stimuli (Xu, 2005; Harley et al., 2009; Engel et al., 2009) and mediated the formation of category-specific representations (van der Linden et al., 2008). Moreover, the right FFA plays an important role in visual discrimination that can be fine-tuned by experience with other domain categories (Engel et al., 2009). Furthermore, previous studies have consistently reported that the left FuG plays a more prominent role than the right FuG in processing non-face related information (Devlin et al., 2006; Bi et al., 2014; Bilalic et al., 2016). In details, the left FuG was engaged in visual word recognition as a connector between the abstract visual information and higher order properties of the stimulus (Devlin et al., 2006) and not only participated in visual categorization learning but also its activity could be modulated by visual learning (Goold and Meng, 2017). In radiological expertise, left FuG activation made the radiologists more sensitive to radiological images and reliably distinguished between upright and inverted X-rays (Bilalic et al., 2016). Additionally, a prior study found that the activity of the left FuG was positively correlated with participants' perceptual performance (Bi et al., 2014) supporting the pivotal role of the FuG in supporting recognition efficiency (Zhang et al., 2022).

In the current study, we speculated that the left FuG plays a vital role in recognizing the stimuli of radiological images, Furthermore, the short-term extraordinarily high load and repetitive usage of the visual system by the RI group can modify the visual processing of radiological stimuli. The fine-tuned behavioral performance and functional adaptation manifest in the superior ability of recognizing the nodule stimulus and stronger neural reflections in the resting state to make the brain much more efficient in detecting nodule-specific features.

### The decreased DC level of the right OrG in radiologists

Decreased DC values of the right OrG were found in the RI group compared with those of the NC group (Figure 4 and Table 2). The OrG, as an OFC subregion (Rudebeck and Rich, 2018), is responsible for decision-making by primarily adjusting the utilities associated with different sensory stimuli (Lee et al., 2007) and plays a critical role in flexible, outcome-guided behavior (Liu et al., 2020). Decision-making is the process of choosing a particular response and further flexibly modifying cognitive and sensorimotor operations based on an evaluation of potential costs (Lee et al., 2007), which is necessary for expert visual processing. Decision making is part of the object recognition process during image interpretation (Wang et al., 2021). Of note, decision-making ability changes dynamically and continually as experience increases (Lee et al., 2007). Hence, the OFC was activated when the participants faced low-cost situations, such as either passively viewing information or selecting among options (Volz et al., 2006). A previous study on baseball batter expertise also verified that the OFC was responsible for expertise-driven rapid visual decisions (Muraskin et al., 2015). In Kirk's study on aesthetic expertise, the recruitment of the OFC between experts and non-experts suggested that this region was involved in expertise-related reward processing (Kirk et al., 2009). A study based on a chess model reported that the OFC appeared to be activated in this comparison between experts and novices (Krawczyk et al., 2011). In the current study, we propose that visual experience modulates radiologists' decision-making processes. Specifically, when radiologists face domain-specific options, they need to make decisions by employing many brain resources to recognize radiological stimuli.

### The changed DC level of bilateral SFG in radiologists

Compared with those of the NC group, changed DC values of the bilateral SFG were found in the RI group (Figure 4 and Table 2). Multiple previous studies have shown that the SFG plays important roles in WM (Klingberg et al., 1997; Su et al., 2022). WM is a central mental capacity; it provides the platform for holding and manipulating thoughts and for organizing goal-directed behavior (Miller et al., 2018). WM capacity, which refers to the ability to retain the maximum amount of information, is a vital factor for problem solving and reasoning ability (Westerberg and Klingberg, 2007). The acquisition of visual expertise might improve WM performance (Moore et al., 2006). The neuroimaging study of Haller and Radue (2005) found that the enhanced neuronal activations of the SFG manifested in better WM capability in the process of radiological expertise. Kesler et al. (2011) found significantly increased activation of the SFG in visual tasks, which participated in online monitoring and manipulation of task-related information. Ouellette et al found lower activation of the lateral SFG in trained radiologists while they viewed medical images, suggesting that WM is a crucial component of radiology expertise and more efficient in radiologists (Ouellette et al., 2020). In our current study, different trends in the bilateral SFG showed that the increased DC values in the left SFG and decreased DC values in the right SFG were closely associated with WM when utilizing radiological expertise. Taking the weight of the brain area into consideration, the overall trend of DC values tended to be negative in the right SFG. Therefore, the decreased DC values of the right SFG may demonstrate increased neural efficiency of the WM process, thus enabling the RI group to spend less energy making a judgment and obtaining a good result compared with that of the NC group. Furthermore, we propose that the altered dynamics of the brain network when acquiring radiological expertise might support remodeling of the WM process reflecting more automated encoding and maintenance WM capacity, indicating a more efficient mechanism subserving visual expertise.

### The decreased DC level of left MFG in radiologists

Decreased DC values of the left MFG in the RI group were found compared with values of the NC group in our study (Figure 4 and Table 2). A previous neuroimaging study found that the MFG participated in visual attention based on the model of radiologists (Haller and Radue, 2005). Selective attention can optimize the processing of information, make radiologists rapidly search for a particular “target” in radiographic images and adjust their response to information collected and compared to previously learned reference images (Haller and Radue, 2005; Harley et al., 2009). Attention has an important impact on visual expertise, even in the earliest step of visual processing (Harel, 2016). The left MFG has a crucial role in the dorsal attention network (DAN) and ventral attention network (VAN) to facilitate interactions between the two networks during attentional processing (Briggs et al., 2021). It has been found that the MFG is an important center facilitating attentional processes (Japee et al., 2015). Haller and Radue (2005) found enhanced neuronal activation of the MFG in radiologists compared with that in non-radiologists, suggesting that the MFG participated in the process of radiological expertise and played an important role in attention control. In contrast, the study of Melo et al. (2011) reported lower activation of the MFG in radiologists than non-radiologists when they were observing medical images. The evidence summarized above suggested that short-term experience could adjust the process of attention control to make it more efficient and enable trainees to have more flexibility in manipulating limited attentional resources so that residual resources could be allocated validly to other brain regions supporting more demanding tasks.

### The decreased DC level of bilateral PhG in radiologists

The comparison between groups revealed decreased DC values of the bilateral PhG in the RI group compared with those of the NC group (Figure 4 and Table 2). The PhG is an important center for memory processing (Lin et al., 2021). The acquisition of visual expertise might be accompanied by the alteration of memory representations (Annis and Palmeri, 2019). Existing neuroimaging studies have reported that chess experts and expert archers recruited more activation of the PhG when responding to domain-specific stimuli (Bilalić et al., 2010; Kim et al., 2011). In our study, the deceased DC values of the bilateral PhG may reflect the highly efficient process of memory encoding and extraction. Short-term experience may contribute to radiology interns spending less energy on employing memory resources when radiologists interpret the radiological images.

### Limitation

It is important to note the limitations of our study. Firstly, the sample size was relatively small, which could limit the generalizability of the results. Future studies with larger sample sizes are needed to confirm the current findings. Secondly, the training duration for radiology interns was relatively short. Although the number of training cases for each participant was sufficient to acquire expertise, a longer training duration could potentially lead to different results. Therefore, future studies should consider longer training periods. Finally, a cross-sectional design was used in this study, which may limit the interpretation of the findings. Longitudinal studies are needed to better understand how visual experience affects brain dynamics in radiologists. Additionally, confounding factors such as long-term experience or congenital factors could have influenced the results. Therefore, future studies should consider controlling for these factors or using a longitudinal design to better understand the effects of visual experience on brain dynamics.

## Conclusions

In conclusion, our findings suggest that visual experience can modulate the dynamics of the resting-state brain network, as reflected in multidimensional neurobehavioral components based on the expertise model of radiologists. These components are strongly interlinked with high-order cognitive and low-order visual factors, including attention control, memory, WM, decision making, and visual processing. These results provide a novel insight into the neural mechanism underlying visual expertise. Despite the limitations of our study, we believe that our findings contribute to the current understanding of how real-world visual experience affects brain activity and may have implications for radiologist training and clinical practice. Further research is needed to confirm and extend our findings.

## Data availability statement

The original contributions presented in the study are included in the article/supplementary material, further inquiries can be directed to the corresponding authors.

## Ethics statement

The studies involving human participants were reviewed and approved by First Affiliated Hospital of Medical College in Xi'an Jiaotong University Subcommittee on Human Studies. The patients/participants provided their written informed consent to participate in this study. Written informed consent was obtained from the individual(s) for the publication of any potentially identifiable images or data included in this article.

## Author contributions

Study design and interpretation of results: CJ and MD. Data collection or acquisition: HW, CJ, and MD. Statistical analysis: MD and XZ. Drafting the manuscript work: HW and RY. Essay revision: JW, CC, CJ, and MD. All authors read and approved the final version of the manuscript and agreed to publish.
