# Corrigendum: Visual expertise modulates resting-state brain network dynamics in radiologists: a degree centrality analysis

**DOI:** 10.3389/fnins.2023.1241073

**Published:** 2023-07-07

**Authors:** Hongmei Wang, Renhuan Yao, Xiaoyan Zhang, Chao Chen, Jia Wu, Minghao Dong, Chenwang Jin

**Affiliations:** ^1^Department of Radiology, First Affiliated Hospital of Xi'an, Jiaotong University, Xi'an, China; ^2^Department of Medical Imaging, Inner Mongolia People's Hospital, Hohhot, China; ^3^Department of Nuclear Medicine, Inner Mongolia People's Hospital, Hohhot, China; ^4^Engineering Research Center of Molecular and Neuro Imaging of Ministry of Education, School of Life Science and Technology, Xidian University, Xi'an, China; ^5^PLA Funding Payment Center, Beijing, China; ^6^School of Foreign Languages, Northwestern Polytechnical University, Xi'an, Shaanxi, China; ^7^Xi'an Key Laboratory of Intelligent Sensing and Regulation of Trans-Scale Life Information, School of Life Science and Technology, Xidian University, Xi'an, China

**Keywords:** degree centrality, visual expertise, object recognition, Support vector machine, radiologist

In the published article, there was an error in the legend for Figure 1 as published, G, H was incorrectly written as D–H.

The corrected legend appears below.

The pipeline of the rs-MRI data analysis. **(A–C)** The resting-state MRI data were collected and preprocessed following procedures described in the Methods. Then, the DC for each voxel was calculated and used for future feature selection. **(D–F)** Feature selection. Two-step feature selection was performed and the first level used a two-sample approach to perform the regional average feature. Then, RFE-SVM modeling with LOOCV was employed to search for the most remarkable features between groups. **(G, H)** SVM modeling. Reliable SVM classification results and the brain areas with robust differences in DC values between groups were obtained to reflect the alteration of dynamics in the whole-brain network. rs-MRI, resting-state MRI; fMRI, functional magnetic resonance imaging; DC, degree centrality; RFE-SVM, recursive feature elimination-support vector machine; SVM, support vector machine; LOOCV, leave-one-out cross-validation.

The authors apologize for this error and state that this does not change the scientific conclusions of the article in any way. The original article has been updated.

